# Pan-Cancer Molecular Patterns and Biological Implications Associated with a Tumor-Specific Molecular Signature

**DOI:** 10.3390/cells10010045

**Published:** 2020-12-31

**Authors:** Darío Rocha, Iris A. García, Aldana González Montoro, Andrea Llera, Laura Prato, María R. Girotti, Gastón Soria, Elmer A. Fernández

**Affiliations:** 1 Facultad de Ciencias Exactas, Físicas y Naturales, Universidad Nacional de Córdoba, Córdoba X5000HUA, Argentina; rocha.dario.bio@gmail.com (D.R.); aldana.gonzalez.montoro@unc.edu.ar (A.G.M.); 2 Centro de Investigación y Desarrollo en Inmunología y Enfermedades Infecciosas, Consejo Nacional de Investigaciones Científicas y Técnicas, Universidad Católica de Córdoba, Córdoba X5016DHK, Argentina; agarcia@cidie.ucc.edu.ar; 3 Departamento de Bioquímica Clínica, Facultad de Ciencias Químicas, Universidad Nacional de Córdoba, Córdoba X5000HUA, Argentina; gaston.soria@unc.edu.ar; 4 Facultad de Matemática, Astronomía y Física, Universidad Nacional de Córdoba, Córdoba X5000HUA, Argentina; 5 Laboratorio de Terapia Molecular y Celular—Genocan, Fundación Instituto Leloir, Consejo Nacional de Investigaciones Científicas y Técnicas, Buenos Aires C1405BWE, Argentina; allera@leloir.org.ar; 6 Instituto Académico Pedagógico de Ciencias Básicas y Aplicadas, Universidad Nacional de Villa María, Villa María, Córdoba X5900, Argentina; l_prato@hotmail.com; 7 Laboratorio de Inmuno Oncología Traslacional, Instituto de Biología y Medicina Experimental, Consejo Nacional de Investigaciones Científicas y Técnicas, Buenos Aires C1428ADN, Argentina; mgirotti@dna.uba.ar; 8 Centro de Investigaciones en Bioquímica Clínica e Inmunología, Consejo Nacional de Investigaciones Científicas y Técnicas, Córdoba X5000HUA, Argentina

**Keywords:** tumor-agnostic classification, PAM50, uncertainty assessment, gene expression, cell-cycle

## Abstract

Studying tissue-independent components of cancer and defining pan-cancer subtypes could be addressed using tissue-specific molecular signatures if classification errors are controlled. Since PAM50 is a well-known, United States Food and Drug Administration (FDA)-approved and commercially available breast cancer signature, we applied it with uncertainty assessment to classify tumor samples from over 33 cancer types, discarded unassigned samples, and studied the emerging tumor-agnostic molecular patterns. The percentage of unassigned samples ranged between 55.5% and 86.9% in non-breast tissues, and gene set analysis suggested that the remaining samples could be grouped into two classes (named C1 and C2) regardless of the tissue. The C2 class was more dedifferentiated, more proliferative, with higher centrosome amplification, and potentially more TP53 and RB1 mutations. We identified 28 gene sets and 95 genes mainly associated with cell-cycle progression, cell-cycle checkpoints, and DNA damage that were consistently exacerbated in the C2 class. In some cancer types, the C1/C2 classification was associated with survival and drug sensitivity, and modulated the prognostic meaning of the immune infiltrate. Our results suggest that PAM50 could be repurposed for a pan-cancer context when paired with uncertainty assessment, resulting in two classes with molecular, biological, and clinical implications.

## 1. Introduction

Molecular classification of cancer has been a central topic for decades, because it generates grounds for biological research and is directly linked to the development of specific therapies for distinct subtypes—a key element of precision medicine [1]. Cancer stratification has traditionally been structured in a tissue-based manner; however, pan-cancer approaches that define subtypes irrespective of their tissue of origin can improve our understanding of the central biological features driving cancer [2], and may enable the extension of known therapeutic approaches from one cancer type to others. For example, tumor-agnostic biomarkers such as micro-satellite instability, oncogenes like NTRK1, NTRK2, and NTRK3, and the recently United States Food and Drug Administration (FDA)-approved tumor mutation burden, are used for tumor-agnostic patient stratification and therapeutic decisions. Cancer molecular classification is one of the main goals of The Cancer Genome Atlas (TCGA) project [3], which offers thousands of tumor samples from multiple cancer types that enable exploring shared molecular patterns and stratification criteria. In line with the TCGA goals, it becomes crucial to study whether molecular profiles can effectively categorize samples regardless of the tissue of origin, to determine if molecular subtypes can be delineated to disentangle tissue-specific from tissue-independent components of disease, and also to evaluate if there are predictive expression-based signatures for genomic events that transcend tissues. Facing such inquiries may help in the design of new strategies—or in the repurposing of existing ones—to stratify and treat patients.

In the search for molecular classification criteria able to subdivide any cancer type into relevant subtypes, FDA-approved molecular signatures initially developed for a particular tissue, like PAM50 [4,5], may serve as a starting point and speed up the process of obtaining a kit useable in the clinic. PAM50 is a well-known molecular signature that classifies breast cancer samples into luminal A (LumA), luminal B (LumB), basal-like (Basal) and Her2-enriched (Her2e) subtypes. Interestingly, this breast cancer classification has shown parallelisms with the molecular landscape observed in bladder, pancreatic, lung, and ovarian cancer [6,7,8,9,10,11]. Moreover, PAM50 has been used to classify diverse carcinomas into Basal, LumA, and LumB subtypes, revealing a luminal/basal contrast with biological and potential translational relevance [12]. All those findings suggest that tissue-specific molecular signatures may be useful to reveal tissue-independent components of cancer development and progression, and that PAM50 is a particularly interesting candidate.

Despite those results, there are several shortcomings in the previous uses of PAM50. First, it was applied without uncertainty assessment even though it may misclassify around 30% of breast cancer samples [13], which could result in biased or spurious results. Second, Her2e subtype was dismissed even though ERBB2 has been reported as a factor with therapeutic implications in ovarian, gastric, and esophageal cancer [14]. Moreover, Her2e has a distinctive profile independent of ERBB2 amplification [15], suggesting its relevance even in cancers lacking ERBB2 enrichment. Last, PAM50 has only been applied to epithelial tissues assuming that it reflects their natural luminal/basal epithelial organization [16]; however, its relevance to non-epithelial tumors has not been explored.

We aimed to evaluate if tissue-specific molecular signatures can be applied in a pan-cancer context and reveal broad molecular patterns with biological and clinical relevance that transcend tissues. Specifically, we used the PAM50 signature with an uncertainty assessment method to build a tumor-agnostic classification criterion that was associated with well-known cancer processes, prognosis, and drug sensitivity, and modulated the prognostic ability of the immune infiltrate.

## 2. Methods

### 2.1. Study Design

First, primary tumor samples of 33 different cancer types (including non-epithelial tissues) from TCGA were confidently classified into the LumA, LumB, Her2e, and Basal subtypes. Second, gene set analysis was used to study the pan-cancer differences between those subtypes. The results prompted us to propose a new classification based on PAM50 and permutation-based confidence for molecular classification (PBCMC) [13,17]. Third, the proposed classification was validated on 1449 tumor samples of six cancer types from Gene Expression Omnibus (GEO) [18,19] and on 1018 cell line profiles from the Genomics of Drug Sensitivity in Cancer project (GDSC) [20]. Finally, and serving as further validation, the proposed classification was characterized in all databases in terms of pathways, gene expression, survival, drug sensitivity, and interaction with the immune infiltrate.

### 2.2. Data Acquisition and Pre-Processing

TCGA data were downloaded with TCGA assembler [21], and raw counts and transcripts per million (TPM) were obtained for expression data. Normalization factors for raw counts data were calculated by trimmed mean of M-values (TMM) with EdgeR package [22]. Raw counts expression data were transformed to $log_{2}\left(counts\;per\;million+0.5\right)$ (CPM) when required, as specified in the following sections. Cancer types were abbreviated according to TCGA study abbreviations (Table S1). GEO data were downloaded with GEOquery package [23], features without entrez identifier were removed, repeated entrezids were averaged, and quantile normalization was applied. GDSC expression data were downloaded as Robust Multi-array Average (RMA) normalized expression data for cell lines. Annotation provided as symbol was converted to entrezid with the org.Hs.eg.db package [24], features without valid entrezid were removed, and duplicated entrezids were averaged. GDSC cell lines were split into cohorts according to TCGA cancer type classification (Table S2). Cell lines without available classification and cohorts with less than 4 cell lines were removed. Drug screening data was obtained from GDSC1 and GDSC2 datasets.

### 2.3. Sample Classification and Immune Infiltrate Estimation

The normalized and transformed CPM data was used for classification purposes. The PAM50 algorithm and uncertainty assessment analysis was performed with the PBCMC R package [17] using the standard settings, and samples with low classification certainty (hereafter “unassigned samples”) were removed from further analyses to prevent spurious or biased results. TPM data were used to estimate the immune infiltrate using MIXTURE algorithm [25] with LM22 signature [26].

### 2.4. Gene Set Enrichment and Differential Gene Expression

Two kinds of gene set analysis were performed: Gene Set Variation Analysis (GSVA) [27] and the Massive and Integrative Gene Set Analysis (MIGSA) [28]. GSVA calculates an enrichment score for each gene set in each sample, resulting in data that can be explored with dimensional reduction analysis like t-distributed Stochastic Neighbor Embedding (t-SNE) [29]. MIGSA combines over-representation and functional scoring gene set analysis, resulting in a *p*-value for each gene set, for each comparison (basal against Her2e, basal against luminal A, basal against luminal B, etc.), in each cohort. The use of MIGSA was restricted to cohorts with at least eight samples per subtype in each comparison, following the author´s suggestion. For both methods, a total of 1195 gene sets with lengths between 15 and 500 genes from MSigDB [30] were tested (50 for KEGG [31], 188 for Oncogenic pathways [30] and 957 for Reactome [32]). Expression data were filtered to discard genes that had less than 1 CPM in more than half of the samples within each cohort. For TCGA, raw count data (not counts per million) were used for MIGSA and GSVA algorithms. The simple enrichment analysis (SEA) parameters of MIGSA were adjusted for each cohort to achieve a percentage of differentially expressed genes of approximately five percent. The gene set *p*-values obtained with MIGSA were adjusted with Benjamini andand Hochberg correction within each cohort. The differential gene expression analysis was performed with limma package [33] with log fold change = 0, using voom function for RNA-seq count data with TMM normalization factors, and adjusting the resulting *p*-values with Benjamini andand Hochberg method.

### 2.5. Signatures

Gene signatures were used with entrez gene identifiers when available, else entrezids were obtained from gene symbols using org.Hs.eg.db package. Genes without a valid entrezid were discarded. Signature scores were calculated according to Equation (2), except for the CA20 signature scores which were calculated using Equation (3). Wilcoxon signed-rank test was used to test the significance of the differences between classes, using Benjamini and Hochberg correction for multiple testing within each database. Tumor-normal differences were studied on TCGA participants with tumor and normal paired samples, calculating for each participant and signature a difference according to Equation (1). Tumor-normal differences between classes were tested with Wilcoxon signed-rank test, and Benjamini and Hochberg correction was performed within each database.

### 2.6. Equations

To quantify the difference between tumor and normal samples, a general equation was used in all instances:

(1) $$D{\left(T,\;N\right)}_{p}=\;V_{pt}-\;V_{pn}$$

where $D{\left(T,\;N\right)}_{p}$ is the difference between the tumor and the normal samples, and $V_{pt}$ and $V_{pn}$ are the signature values ($V_{s}$) of participant “*p*” for the tumor “*t*” and normal “*n*” samples, respectively.

The signature value (except for the CA20 signature) for each sample was calculated as the average of the normalized GSVA scores or normalized gene expression:

(2) $$V_{s}=\;\frac{\sum_{i=1}^{k}e_{si}}{k}$$

where $V_{\left(s\right)}$ is the signature value for sample “*s*”, $e_{si}$ is the z-score (normalized) value of the “*i*-th” GSVA gene set score or expression of the “*i*-th” gene for sample “s”, and *“k”* represents the number of genes or gene sets in the signature.

The CA20 signature value for each sample was calculated as the sum of the expression of the signature genes, centered by the median expression of the cohort and scaled by the standard deviation of the cohort:

(3) $$V_{s}=\;\frac{\sum_{i=1}^{k}e_{sg}-m}{\sigma }$$

where $V_{\left(s\right)}$ is the signature value for sample “*s*”, $e_{g}$ is the expression of gene “*g*” from the signature in sample “*s*”, “*m*” is the median expression of all the genes in the cohort, “σ” is the standard deviation of the expression of all the genes in the cohort, and “*k*” is the number of genes in the signature.

### 2.7. Survival Analysis

Cox proportional hazards models were used. Considering that most randomly generated molecular signatures are associated with survival in breast and other cancer types [34,35], the performance of a classification needs to be compared to the performance of classifications based on randomly selected genes. To generate classifications based on random selection of genes, first, a set of 50 genes was randomly selected from the whole pool of genes; second, a principal component analysis was performed using the genes z-scores, and the samples were divided into two categories using as cut point the median of the first principal component; and third, a random subset of samples was selected so that the final number of samples matches the number of samples in the binary classification under study. That process was repeated 5000 times for each cohort, and we use the term “V-score” to refer to the proportion of those repetitions that showed a more prominent hazard ratio and smaller *p*-value than the ones observed with the classification under study.

### 2.8. Drug Analysis

A simple lineal model was adjusted for each drug in each cancer type to model the $\ln\left(IC_{50}\right)$ as a function of class (C1/C2). Within each cancer type, *p*-values were adjusted with Benjamini and Hochberg correction. Shapiro–Wilk’s and Levene’s tests were used in each model to check the model assumptions. Model *p*-values were adjusted within each cohort using Benjamini and Hochberg method. Only drugs with adjusted model *p*-value < 0.01, Shapiro–Wilk’s *p*-value > 0.05, and Levenne’s test > 0.05 were considered significantly different between classes.

## 3. Results

### 3.1. Two Molecular Classes Emerge in All Tissues

PAM50 combined with PBCMC was used on TCGA cohorts, discarding unclassified samples in order to work only with good representatives of the subtypes (i.e., confidently classified samples). The proportion of unclassified samples was 26% for breast cancer in accordance to previous studies [13], and between 55.5% to 86.9% (median 71%) for the remaining cancer types (Table S3). Such high proportions of unclassified samples suggest that using a tissue-specific molecular signature in other tissues needs an uncertainty assessment method in order to prevent spurious results. Interestingly, gynecological cancer types (CESC, UCEC, OV, and UCS) that have been clustered with BRCA based on a pan-cancer multi-omic approach [36] had high percentages of unclassified samples (86.9%, 68.9%, 75.5%, and 86%, respectively), demonstrating that the proportion of unclassified samples is not necessarily associated with molecular similarity.

**Figure 1:**
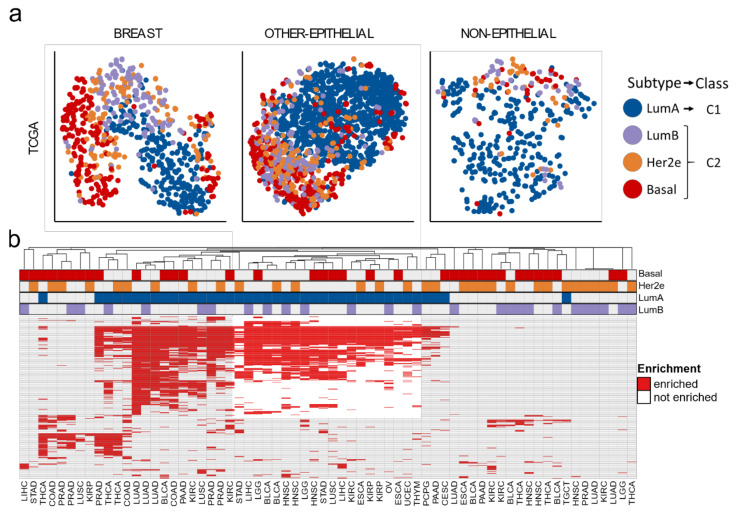
PAM50 classification across The Cancer Genome Atlas (TCGA) cohorts. (**a**) T-distributed Stochastic Neighbor Embedding t-SNE projection of Gene Set Variation Analysis (GSVA) individual sample scores. Scores were normalized within each cohort. (**b**) Massive and Integrative Gene Set Analysis (MIGSA) results comparing PAM50 subtypes to one another. Each column contains the cohort results indicated in the bottom row and the pair of subtypes indicated with colors at the top. Each row corresponds to a particular gene set. Enriched gene sets are those with *p*-adjusted < 0.05.

We then compared the four PAM50 subtypes (LumA, LumB, Her2e and Basal) to one another using the MIGSA and GSVA methods to search for pathways that transcend tissues. We chose the gene set analysis approach instead of differential gene expression because this approach can account for the genetic heterogeneity between cohorts, providing more concordant results in this pan-cancer scenario [28,37,38]. The GSVA results in TCGA data suggest that PAM50 subtypes can be grouped into two major classes (Figure 1a), where LumA samples can be distinguished from the rest of the subtypes, while LumB, Her2e, and Basal tend to overlap with each other. These two classes can also be observed in non-epithelial cancer types, proving that PAM50 classification does not necessarily reflect the luminal/basal organization of epithelial tissues as reported in another study [16]. The same two classes emerged from MIGSA results (Figure 1b), because a group of gene sets consistently differentiates LumA from any of the other subtypes irrespective of the cohort, and no group of gene sets differentiates LumB, Her2e, or Basal from each other consistently across cohorts.

Therefore, in TCGA data, both GSVA and MIGSA suggest that the samples classified with PAM50 and uncertainty assessment fall into two major classes: C1 comprising LumA samples, and C2 comprising LumB, Her2e, and Basal samples. The consistency of the results across tumor types encouraged us to propose a classification strategy based on PAM50 and uncertainty assessment: first apply PAM50 with an uncertainty assessment algorithm (PBCMC) discarding samples with low classification certainty, then LumA samples constitute the C1 class, and LumB, Her2e, and Basal constitute the C2 class.

**Figure 2:**
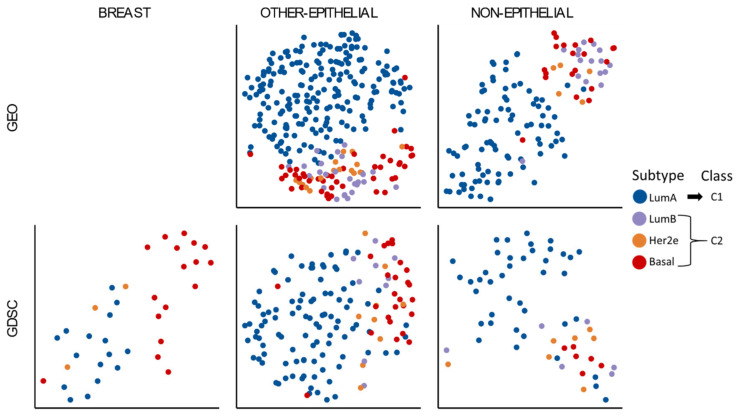
PAM50 classification across Gene Expression Omnibus (GEO) and Genomics of Drug Sensitivity in Cancer (GDSC). GSVA individual sample scores were standardized within each cohort and displayed using a t-SNE projection.

This classification proposal obtained on TCGA cohorts was then validated on GEO and GDSC datasets. The selected cancer types from GEO were bladder (GSE48075 [6]), colon (GSE39582 [39]), and gastric cancer (GSE66229 [40]) as epithelial types; and follicular lymphoma (GSE127462 [41]), glioblastoma multiforme (GSE122586 [42]), and uterine sarcoma (GSE119041 [43]) as non-epithelial types. We did not use MIGSA at this stage due to the limited number of samples (less than 8 in each subtype). The GSVA enrichment scores for individual samples were calculated and analyzed with t-SNE dimensional reduction, and the results resembled the TCGA results (Figure 1a): LumA samples seem to constitute one group, while the union of LumB, Her2e, and Basal samples seem to represent another group (Figure 2). This was observed even in non-epithelial cancer types, confirming that the two proposed classes (rather than the four PAM50 subtypes) seem to be identifiable in a pan-cancer context. The proposed classification for all the samples and cell lines included in this study can be found in Table S4.

### 3.2. Cell-Cycle Checkpoints and Progression Pathways Drive the Differences between C1 and C2 Classes

**Figure 3:**
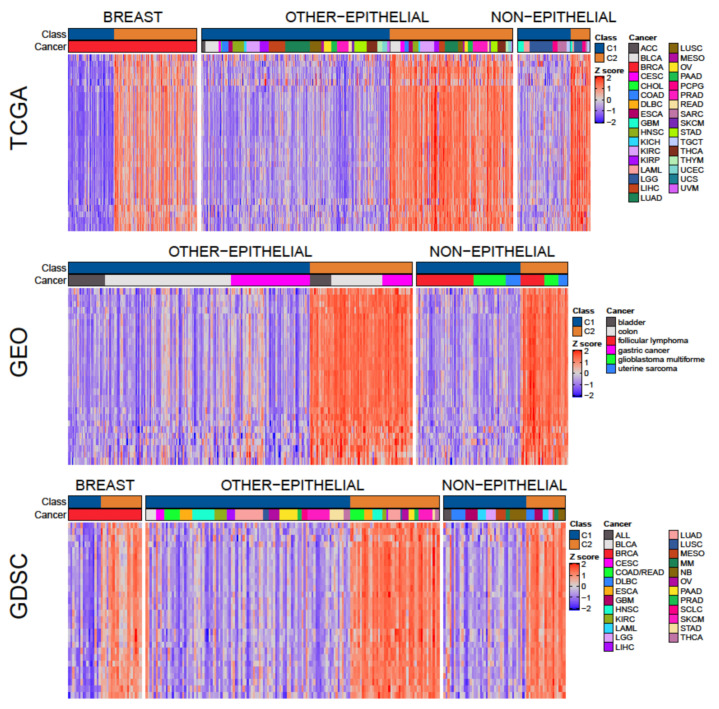
Gene set analysis of C1 and C2 classes across cohorts. The heatmaps show the standardized GSVA enrichment score of each sample (in columns) for each of the 28 selected gene sets (in rows), in cohorts from TCGA, GEO, and GDSC.

To validate the proposed classes, they were studied at the gene set level using MIGSA to compare C1 with C2 in 27 cohorts from TCGA and 6 from GEO (only cohorts with at least 8 samples in each class were used). Out of the 1195 gene sets analyzed, 126 were differentially enriched between C1 and C2 in more than half of the studied cohorts, with 28 gene sets differentially enriched in at least 30 out of the 33 cohorts (Table S5). Therefore, these 28 gene sets represent key functional differences between C1 and C2 in most cancer types. Since not all cohorts could be studied with MIGSA, GSVA was used on all cohorts to explore enrichment patterns. The GSVA enrichment scores of the 28 selected gene sets were consistently higher in C2 than in C1 across all cohorts (Figure 3), evidencing the existence of these two classes in all cancer types. Most of those 28 gene sets are associated with cell-cycle checkpoints and progression. For example, the gene sets *G2/M checkpoint*, *activation of ATR in response to replication stress*, and *mitotic spindle checkpoint* indicate alterations in pathways associated with cell-cycle-arrest in response to DNA damage, which are central alterations in cancer [44]. Other gene sets, like *resolution of sister chromatid cohesion*, *E2F targets*, *polo-like kinase mediated events*, and *mitotic G1 phase* and *G1/S transition* indicate alterations in pathways related to cell-cycle progression.

**Figure 4:**
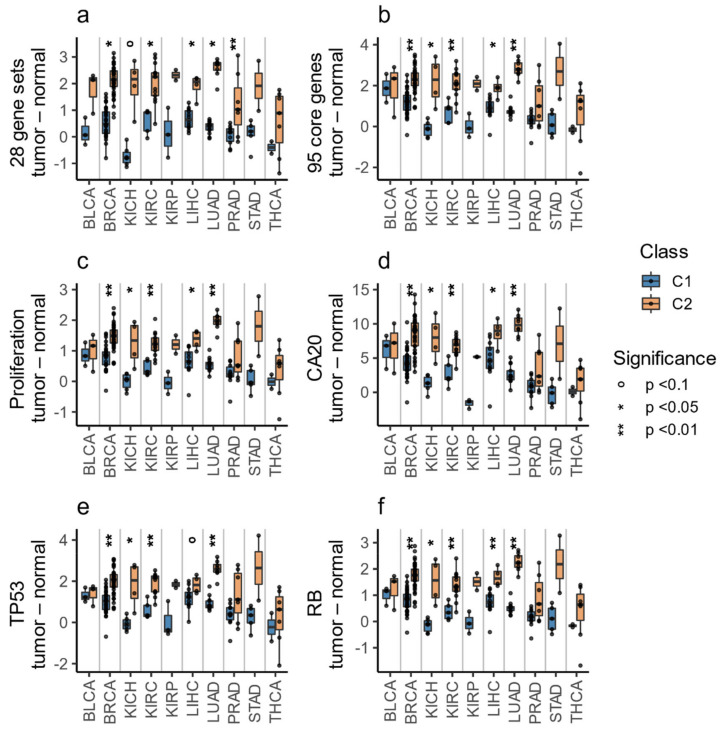
Tumor-normal differences in TCGA participants. Each dot represents the difference between a tumor sample and its paired normal tissue sample, according to the average GSVA enrichment score of the 28 selected gene sets (**a**), the average expression of the 95 core genes (**b**), or the scores for the proliferation, CA20, TP53, and RB signatures (**c**–**f**). The *p*-values were obtained with the Wilcoxon signed rank-test.

Cell-cycle checkpoints and cell-cycle progression pathways are closely associated with proliferation, and together they represent some of the most fundamental differences between normal cells and cancer cells [45,46]. Since these features differentiate C1 from C2, we wondered which class presents a molecular profile more similar to normal cells. To address this question, we used TCGA participants with paired tumor and normal tissue samples, restricting the analysis to cohorts with at least two samples in each class—resulting in 10 cohorts. For each participant, the difference between the tumor and normal sample was calculated (methods: Equation (1)), where the value for each sample was the average of the 28 normalized GSVA scores of the selected gene sets (methods: Equation (2)). In all analyzed cohorts, the tumor-normal difference in the C1 class was smaller than in the C2 class (Figure 4a) and was significant in five cohorts (Wilcoxon signed rank test with Benjamini and Hochberg correction for multiple comparisons, *p* < 0.05). Therefore, compared to normal tissue, the C2 class presented a greater difference between normal and tumor samples in terms of cell-cycle checkpoints, cell-cycle progression, and response to DNA damage pathways, which suggests a more aggressive and proliferative profile [47].

### 3.3. A Tumor-Agnostic Set of Genes Differentiate C1 from C2 Classes

**Figure 5:**
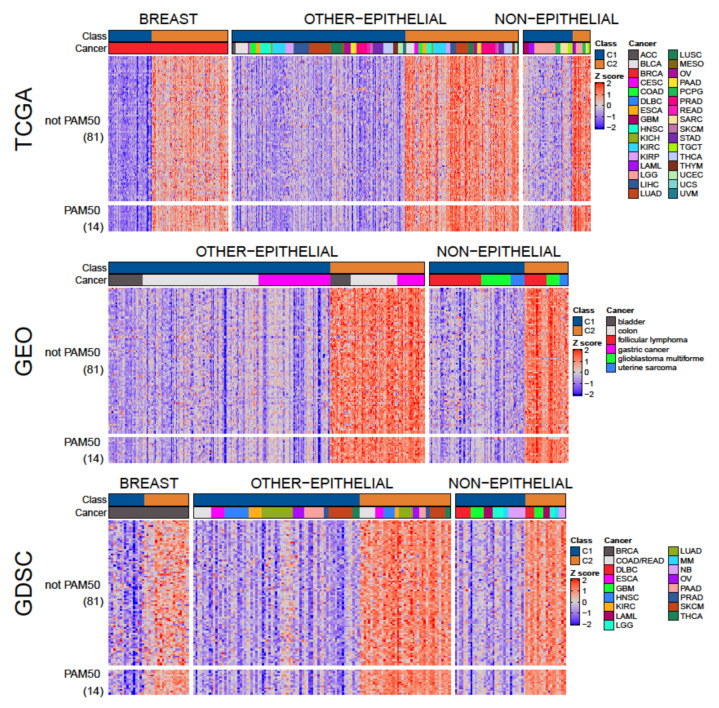
Expression of the 95 core genes in C1 and C2 classes across cohorts. Genes are arranged in rows and samples in columns. Expression values are standardized within each cohort.

To characterize the C1 and C2 classes at the gene expression level, we performed a differential gene expression analysis on each cohort to compare these classes. In order to be consistent with the pathway analysis, only cohorts with at least eight samples in each class were used, resulting in 33 cohorts (27 from TCGA and 6 from GEO). The number of genes present in all TCGA, GEO, and GDSC databases was 8364, 1273 of which were differentially expressed in at least half of the analyzed cohorts (Table S6), and 95 were significantly more expressed in C2 than C1 in 30 out of the 33 cohorts (Table S6, Figure 5). Among these 95 genes, only 14 are used by the PAM50 signature, which are among the 21 genes described by Parker as separating luminal A from the other subtypes in breast cancer [4]. The differences observed in the 33 analyzed cohorts could also be observed in the remaining TCGA and GDSC cohorts (Figure 5), showing that these 95 genes represent the core differences between C1 and C2 classes in most cancer types, and, in line with the gene set results, they are mostly enriching retinoblastoma, cell-cycle, DNA replication, and DNA damage response pathways (Table S7).

The differences between tumor and normal samples in terms of the 95 core genes were studied similarly to what was explained in the gene set analysis section (methods: Equations (1) and (2)). Analogously to the pathway results, the molecular profile of the C1 class was more similar to normal samples, while the C2 class presented a higher expression of the 95 core genes (Figure 4b).

### 3.4. The C1/C2 Classification Is Associated with Proliferation, Centrosome Amplification, TP53, and Retinoblastoma Pathways

Gene set and gene expression analyses revealed that the main differences between C1 and C2 are associated with cell-cycle progression, cell-cycle checkpoints, and DNA damage response. To further validate and characterize the proposed classification, we studied some well-known biological processes for which we could find previously published molecular signatures.

The differences between C1 and C2 observed so far were closely associated with proliferation, which is probably the most fundamental trait of cancer [46] and an important contributor to treatment response [48]. Therefore, we studied proliferation using an in-vitro derived signature with 110 genes with prognostic power and potential predictive value in breast, renal, and lung cancer [49]. Another significant difference between the C1 and C2 classes is spindle checkpoint pathways, associated with centrosome amplification [50]. This feature can be found in pre-neoplasias and tumors from multiple cancer types, and multiple compounds targeting centrosomal proteins have shown promising results [51]. Therefore, we studied centrosome amplification using a 20 genes signature (CA20) [52] that is associated with unfavorable prognosis in multiple TCGA cohorts [53]. The last major difference between C1 and C2 is associated with response to DNA damage and apoptosis pathways. One iconic protein associated with these processes is the retinoblastoma protein, encoded by the RB1 gene; therefore, we explored this pathway using a pan-cancer signature (RB) with 182 genes that has been derived from in-vitro experiments and validated in multiple cancer types from METABRIC and TCGA [54]. This signature is associated with heterozygous loss and deletion of RB1, loss of CDKN2A, amplification of CCND1, and amplification of CDK4, and is prognostic in ACC, LGG, KIRK, and SARC. Another iconic protein associated with DNA damage response and apoptosis is p53, encoded by TP53; therefore, we studied the TP53 pathway using a downstream transcriptomic signature (TP53) consisting of four genes (CDC20, CENPA, KIF2C, and PLK1). This TP53 signature is consistently up-regulated in TP53-mutated cancers and positively associated with increased chromosomal instability [55].

For each of those signatures, a sample-based score was calculated in cohorts with at least two samples in each class (C1 and C2), which accounts for a total of 56 cohorts: 32 from TCGA, 6 from GEO, and 18 from GDSC. The signature scores were calculated as the average of the normalized expression of the signature genes (methods: Equation (2)), except for the CA20 signature (methods: Equation (3)). To study the difference between normal and tumor samples, we used the same approach explained during gene set and gene expression analysis (Methods: Equation (1)).

**Figure 6:**
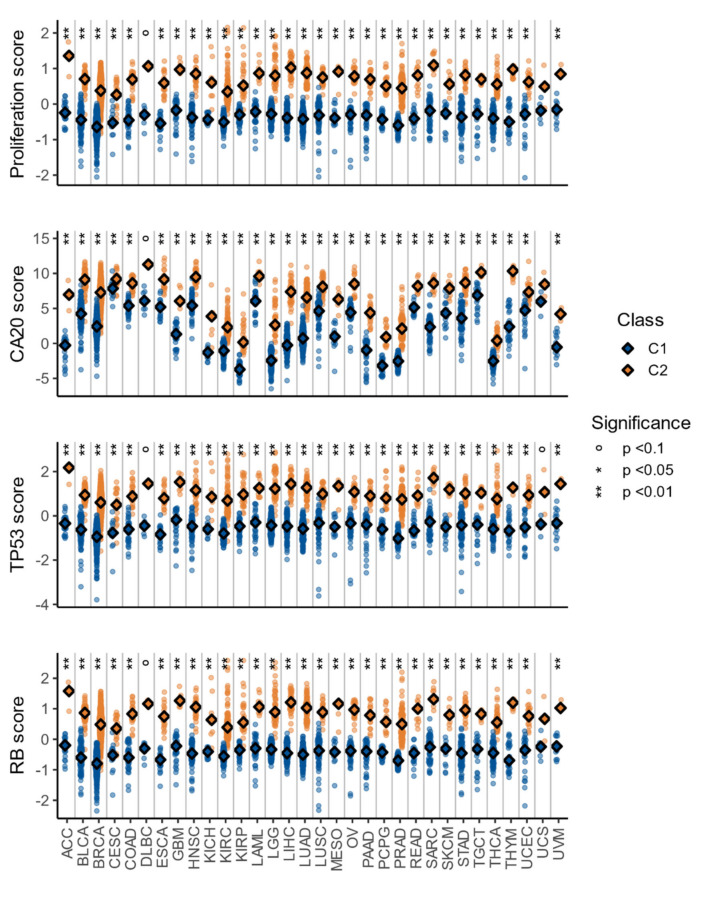
Proliferation, CA20, TP53, and RB signature scores for TCGA samples. The *p*-values were obtained with the Wilcoxon signed rank-test.

**Figure 7:**
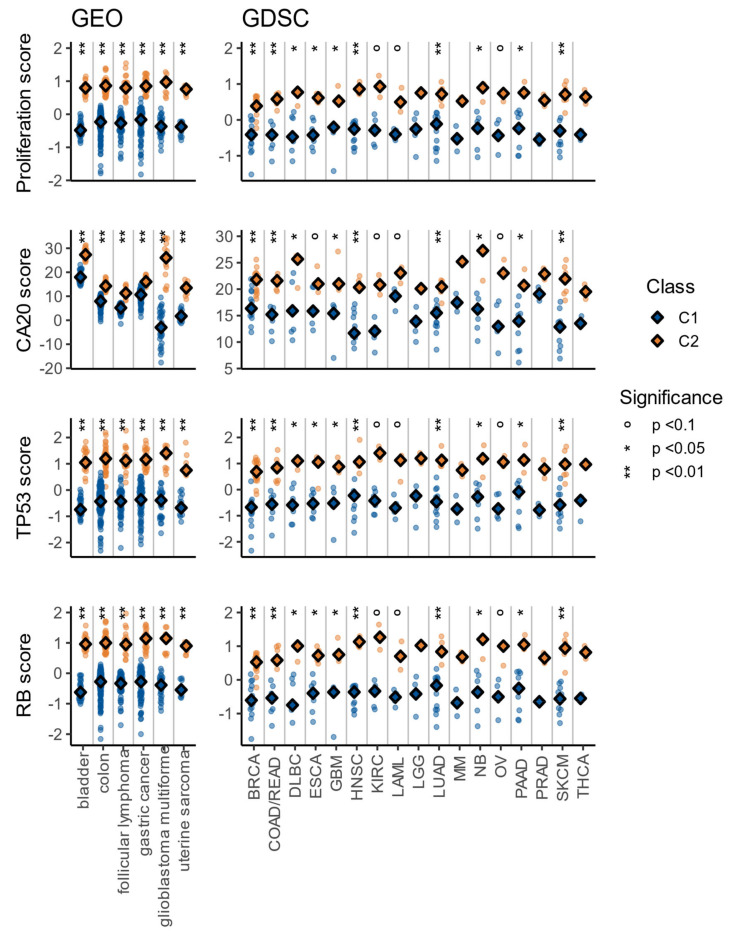
Proliferation, CA20, TP53, and RB signature scores for GEO and GDSC samples. The *p*-values were obtained with the Wilcoxon signed rank-test.

In all cases, the scores were higher in C2 than in C1, with significant results (Wilcoxon signed rank test with Benjamini and Hochberg correction for multiple comparisons, *p* < 0.05) in 30 of the 32 TCGA cohorts (Figure 6), all 6 of the GEO (Figure 7) cohorts, and at least 10 of the 18 GDSC cohorts (Figure 7). Additionally, and in line with pathway and gene expression analysis, the C1 class was more similar to normal tissue (Figure 4c–f). These results indicate that the C2 class is the more proliferative one, with increased chromosomal instability, potentially higher rate of TP53 and RB1 alterations, worse prognosis, and more different to normal tissue.

### 3.5. The C1/C2 Classification Is Associated with Tumor Differentiation and Embryonic Stem Cell-Likeness

**Figure 8:**
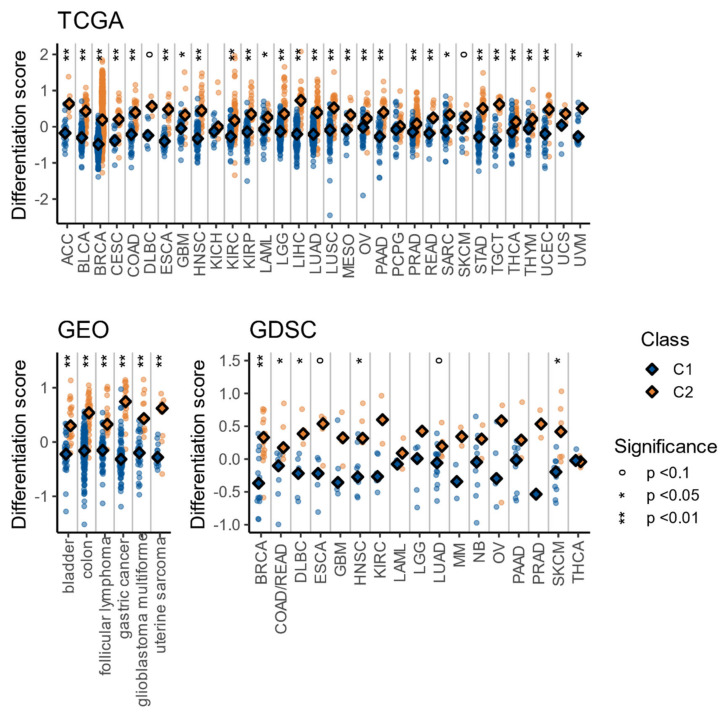
Embryonic Stem Cell-likeness signature scores across all databases (TCGA, GEO, and GDSC). The *p*-values were obtained with the Wilcoxon signed rank-test.

Bearing in mind that the C1 class is comprised entirely of luminal A tumors—which are usually more differentiated than basal tumors in breast cancer [56]—and considering that tumors with higher rates of proliferation, chromosomal instability, and mutations are usually poorly differentiated [57], we suspected that the C2 class may be more dedifferentiated. To assess this, we studied nine genes (ZIC1, TCF7L1, KLF5, MYBL2, NFE2L3, TEAD4, ILF3, HMGA1, and HMGB3), commonly found in embryonic stem cells, that have been found to be overexpressed in poorly differentiated breast, bladder, and glioblastoma tumors [58]. As explained in previous sections, a signature score was calculated for each sample in cohorts with at least two samples in each class (C1 and C2) (methods: Equation (2)). As observed with the other signatures, the score was higher in the C2 class in all cohorts, reaching statistical significance (Wilcoxon signed rank test with Benjamini and Hochberg correction for multiple comparisons, *p* < 0.05) in 27/32 of the TCGA cohorts, 6/6 of the GEO cohorts, and 6/18 of the GDSC cohorts (Figure 8). These results suggest that the C2 class has a more embryonic stem cell-like transcriptomic profile, which correlates with poorer differentiation and worse prognosis [58].

### 3.6. The C1/C2 Classification Is Associated with Patients’ Survival

**Figure 9:**
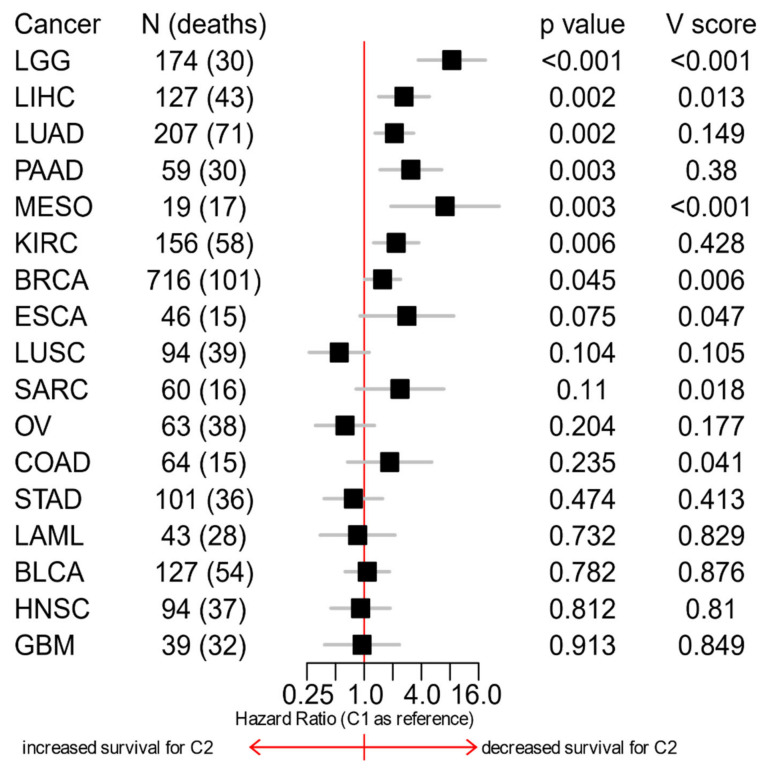
Survival forest plot for TCGA cohorts with at least 10 death events. V-score represents the proportion of random classifications (derived from randomly selected genes) that performed better (in terms of *p*-value and hazard ratio) than the C1/C2 classification.

All the observed differences between the C1 and C2 classes suggest that the C2 class may present a worse prognosis, therefore we explored the prognostic ability of the C1/C2 classification. We only used cohorts with at least 10 death events, and the performance of the C1/C2 classification was compared to the performance of classifications based on random sets of genes. The C1/C2 classification was prognostic in 7 of the 17 analyzed cohorts (univariate Cox regressions, *p* < 0.05): LGG, LIHC, LUAD, PAAD, MESO, KIRC, and BRCA, with the C2 class having a decreased survival (Figure 9), which is consistent with the reports of the proliferation, CA20, TP53, and RB signatures [49,52,53,54,55]. The cohorts where the C1/C2 classification seemed to be more associated with survival were also the ones with lower scores for proliferation [59], CA20 (Figure 6 and Figure 7), TP53 [55], and RB [54] signatures. This phenomenon of proliferation being more prognostic in cancer types with lower indices of proliferation has been reported before for TCGA data [59]. However, that might be exclusive of this database, because a higher Ki67 score—a very well-known pan-cancer proliferation marker with prognostic power [60]—is prognostic in more proliferative cancer types like bladder [61], cervical [62], and ovarian [63] in non-TCGA databases. Therefore, the C1/C2 classification might be prognostic in more cancer types than the ones highlighted in our study. For LGG, LIHC, and MESO, the C1/C2 classification performed better than 98% of the classifications based on random sets of genes, indicating that this classification has a particularly good prognostic ability in those cohorts. For other cohorts, like PAAD, even though the C1/C2 classification had a significant prognostic power, there was a high proportion of random classifications that outperformed it. That does not invalidate the biological meaning of the classification, but it suggests that, in those cohorts, there is a very high number of genes associated with survival.

### 3.7. A Few Transcription Factors Could Cause Most of the Differences between C1 and C2 Signatures

The analysis performed so far suggests that C1 and C2 present universal gene expression profiles that describe two general tumor profiles linked to prognosis and well-known cancer processes. Given the consistency of the results across cohorts and the close relationship between the deregulated pathways, we wondered whether key transcription factors could be orchestrating the differences between these classes. To explore that, we used iRegulon [64] to search for the main factors targeting the 95 core genes previously defined. Nine transcription factors were identified (E2F4, SIN3A, E2F1, TFDP1, MYBL2, NFYB, FOXM1, NFYA, and E2F2), each targeting at least 53 of the 95 core genes. Of particular interest are E2F1 and FOXM1, because they control 75 and 57 of the 95 genes and are also more expressed in C2 than in C1 in 30 and 33 cohorts, respectively. Additionally, together they target 87 of the 95 core selected genes, suggesting that these two transcription factors could be largely involved in the molecular differences observed between C1 and C2.

E2F1 and FOXM1 are two well-studied genes involved in cancer. The transcription factor E2F1 is involved in DNA repair and replication in multiple cancer types [65] and an “addiction” to this oncogene is often present [66]. The overexpression of E2F1 in cancer cells can be associated with increased resistance to several chemotherapeutic drugs [67] and antimetabolite drugs that target enzymes involved in DNA synthesis [66]. This transcription factor is a druggable target that has been evaluated in melanoma [68] and ovarian cancer [69], as well as in TP53 and RB1 defective tumors [70], and its downregulation has shown anti-tumor properties in colorectal cancer [71]. Regarding FOXM1, this transcription factor is a master regulator in cancer [72] that is regulated by E2F1 [70], and its overexpression is associated with an increased stem-like cell population and invasiveness in breast cancer [73], which is consistent with the C2 class being more dedifferentiated. This gene is also associated with resistance to radiation and chemotherapy, therefore inhibiting its activity could increase the responsiveness of some patients [74], and it has been suggested as a potential therapeutic target in solid tumors in general [75] and multiple myeloma [76].

### 3.8. Drug Sensitivity Patterns of C1 and C2 Classes

The differences between C1 and C2 in terms of proliferation and expression of E2F1 and FOXM1 transcription factors suggest that these two classes may respond differently to therapy; therefore, we explored the association of the C1/C2 classification with drug or compound sensitivity using two different approaches. 

First, an enrichment analysis of the 95 core genes was performed over the drug signature database DSigDB [77] with Enrichr [78], which resulted in 255 drug sets differentially enriched (Table S8). Given that the 95 core genes are consistently differentially expressed between C1 and C2, these 255 drugs may have a different effect on those classes regardless of the cancer type. The top 3 enriched drugs were etoposide, monobenzone, and trifluridine. Etoposide is a chemotherapy agent used in multiple cancer types [79], while monobenzone combined with imiquimod induces antimelanoma immunity in cutaneous metastases [80], and trifluridine is used in metastatic colorectal cancers that are refractory to other therapies [81]. These results suggest potential repurposing of multiple drugs, showing that a specific subpopulation of some cancer types may be affected by drugs generally used on other cancer types.

**Figure 10:**
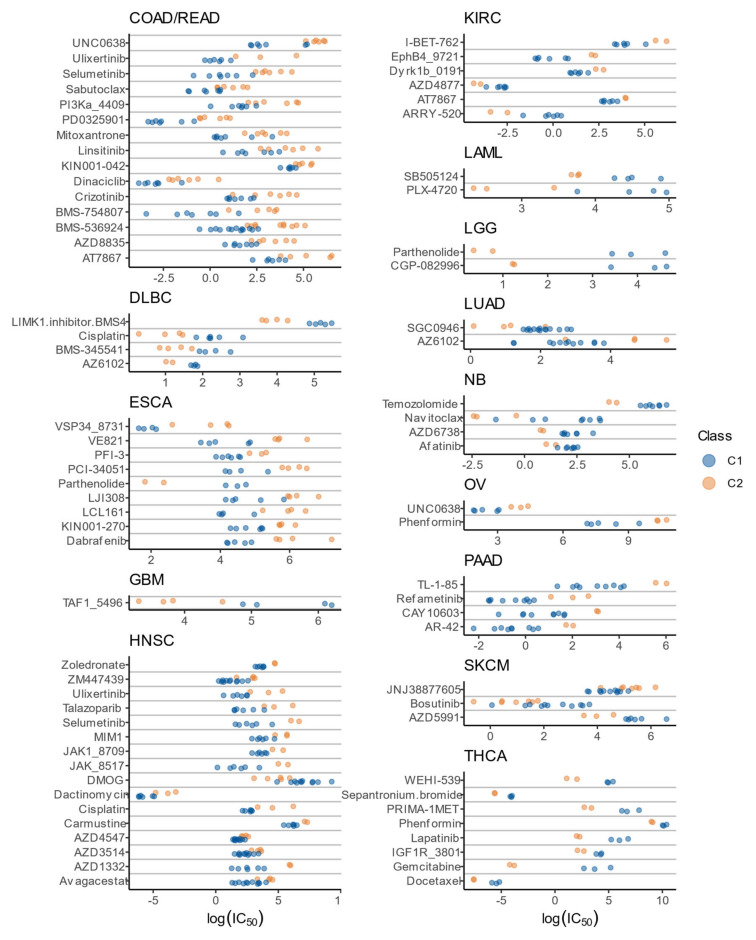
ln(IC_50_) by class for different cell lines. Only significant results (ANOVA, *p* < 0.01) for non-breast cancers are shown.

The second approach was a retrospective analysis of in-vitro assays performed in a collection of cancer cell lines from the GDSC project. Only cohorts with at least two cell lines in each class were analyzed, resulting in a total of 78 drugs (ANOVA, nominal *p* < 0.01) with different IC_50_ values in C1 compared to C2 in non-breast cohorts (Figure 10). The pathways targeted by these drugs are mostly involved in apoptosis regulation, cell-cycle, chromatin histone acetylation/methylation, DNA replication, ERK MAPK signaling, genome integrity, IGF1R signaling, PI3K/MTOR signaling, and RTK signaling (Table S9). Some drugs showed a remarkable difference between classes, like parthenolide in ESCA and LGG (Figure 10). Parthenolide has shown a wide range of biological activities with low toxicity and interesting results as a single therapeutic agent and in multimodal therapies [82]. Our study suggests that the C2 class in ESCA and LGG may be more susceptible to this compound. Beyond the results of individual drugs, the most noteworthy result was that, in general, one class consistently showed increased resistance to most drugs. For example, in DLBC, GBM, LAML, LGG, NB, and THCA, the C2 class was overall more susceptible to all the significant drugs (Figure 10).

### 3.9. The Immune Infiltrate Prognostic Value Is Affected by the C1/C2 Classification

Increasing evidence from preclinical and clinical studies suggests that the outcome of targeted therapeutics against key tumor growth and dissemination factors can be reinforced using immunotherapy approaches in an effective “chemo-immunotherapy” strategy [83]. In this regard, the tumor immune infiltrate is currently under intense research because of its growing prognostic and predictive implications. However, the association between different immune components and survival is not yet fully understood, with numerous cases of immune cell types with contradictory results or different effects according to the varying characteristics of the tumor [84]. For example, the prognostic role of FoxP3(+) Tregs is influenced by tumor site, stage, and molecular subtype [85]. Therefore, there is a need to study the immune infiltrate in combination with other factors rather than as individual cell types. To address this issue, we studied whether the prognostic meaning of the immune infiltrate was affected by the C1/C2 classification.

**Figure 11:**
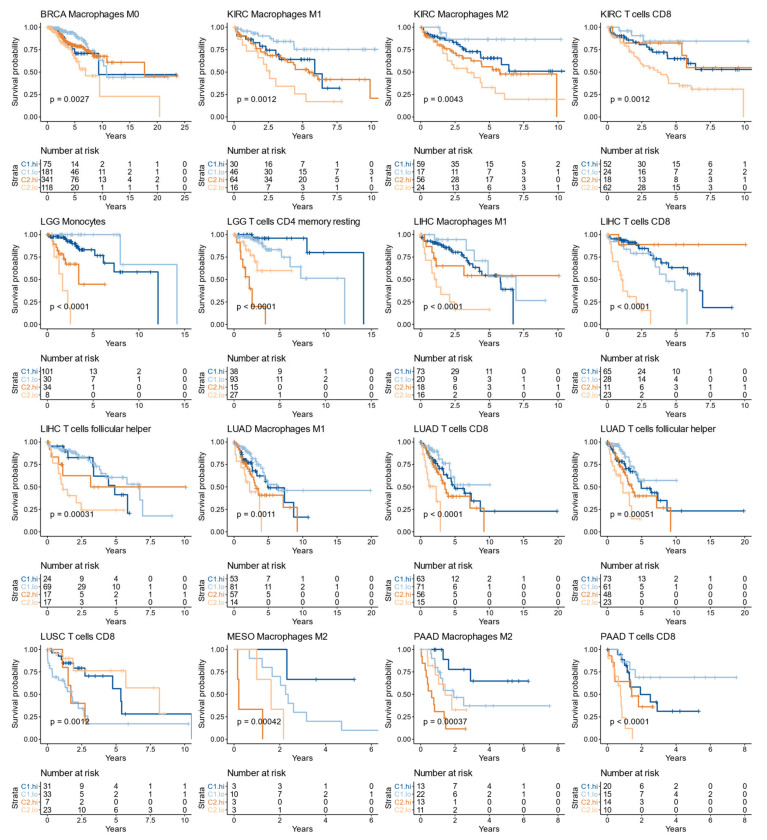
Kaplan-Meier plots for the immune infiltrate (high = hi, low = lo) and C1/C2 class in different TCGA cohorts. *p*-values were obtained with the log-rank test of the complete models. The figure only includes cases where the complete model was significant (Cox regression, log-rank test, *p* < 0.05), the interaction term (class—immune infiltrate) was significant (Cox regression, *p* < 0.05) and the complete model performed better than the univariate model for the immune infiltrate (analysis of Deviance, *p* < 0.05).

In 28 cases we found evidence (Cox proportional hazards model and interaction terms with *p* < 0.05) that the hazard ratio (between the low and high immune infiltrate groups) in the C1 class was different than the hazard ratio in the C2 class (Table S10). For example, in LUSC, biopsies with high CD8 T cells infiltrate showed an improved survival in the C1 class (log-rank test, *p* = 0.012), but a reduced survival in the C2 class (log-rank test, *p* = 0.033) (Figure 11). Despite high CD8 T cells infiltrate usually being associated with increased survival in multiple cancer types [84], here we identified a subpopulation (C2 class) of LUSC samples with the opposite trend. This negative association between CD8 T infiltrate and survival has also been observed in clear cell renal cancer and prostate cancer [84]. Interestingly, even though the C1/C2 classification was not directly associated with survival in LUSC (Figure 11), this classification improved the prognostic ability of the CD8 T cells infiltrate in that cohort (analysis of Deviance, *p* = 0.049). Moreover, the C1/C2 classification improved the prognostic ability of the immune infiltrate in 55 cases from nine cohorts (BRCA, ESCA, KIRC, LGG, LIHC, LUAD, LUSC, MESO, and PAAD), where the complete Cox model (i.e., the model including the C1/C2 classification, the immune infiltrate, and their interaction) performed significantly better (analysis of Deviance, *p* < 0.05) than the univariate model (i.e., the model with only the immune infiltrate as explanatory variable). Therefore, our results suggest that cell-cycle markers, which are closely associated with the C1/C2 classification, can improve the prognostic ability of immune markers. This has been observed in non-small-cell lung carcinoma, where a classification based on proliferation and PD-L1 expression was associated with survival in patients treated with immune checkpoint inhibitors [86]. Overall, the prognostic meaning of cell types tended to differ between classes (Figure 11), and considering the wide variety of emerging markers of response to anti-PD-1 therapy—that even encompasses gut microbiome and body mass index [87]—our results point to cell-cycle progression and checkpoints as interesting factors to study in combination with other markers.

## 4. Discussion

Expression-based signatures that can effectively classify tumor samples independently of their histology may reveal tissue-independent components of cancer that can push forward our knowledge of the disease and help in the design of new therapies and clinical trials. In this sense, molecular signatures originally devised and validated for a single cancer type could provide useful information in a pan-cancer scenario.

This study was focused on the FDA approved breast cancer PAM50 signature paired with an uncertainty assessment method. We subdivided each cancer type into two classes: C1 comprising confidently assigned luminal A samples, and C2 comprising confidently assigned luminal B, Her2e, and Basal samples. This grouping of PAM50 subtypes emerged from a pathway analysis performed on 18 TCGA non-breast cancer types, and it was studied from multiple perspectives in 32 non-breast cohorts from TCGA, 6 cohorts from GEO, and over 1000 cell line profiles from GDSC. This reduction in the number of groups (from four subtypes to two classes) suggests that the genes that PAM50 uses to subdivide the more proliferative tumors into luminal B, Her2-enriched and basal-like (ESR1- and ERBB2-associated genes) [82] do not serve that same purpose in a pan-cancer context. However, those genes can still play a role in the algorithm to distinguish the C1 and C2 classes in at least some cancer types. Another observation is that the PAM50 algorithm did not perform better (in terms of the proportion of confidently assigned samples) in cancer types more related to breast cancer (like gynecological cancer types) than in seemingly unrelated cancer types. This suggests that exploring the relevance of a tissue-specific molecular signature in foreign tissues does not need to be limited to the tissues that are known to be similar to the native one.

The molecular differences between the C1 and C2 classes were remarkably consistent between methods and across cohorts from all different data sources. We identified 28 gene sets and 95 core genes exacerbated in the C2 class, and two transcription factors (E2F1 and FOXM1) that are known to affect prognosis and response to therapy in different cancer types, and might be responsible for most of the differences between the two classes. The C1/C2 classification was also closely associated with multiple molecular signatures that describe other well-known cancer processes such as centrosome amplification, retinoblastoma protein pathway, and differentiation. Therefore, this study shows that a tissue-specific molecular signature can be used in a pan-cancer context and reveal components of the disease that have the same biological meaning regardless of the cancer type.

We additionally studied the prognostic relevance of the C1/C2 classification, finding several cancer types where the C2 class had a significantly shorter survival. Even when not directly prognostic on its own, the C1/C2 classification modulated the prognostic ability of the immune infiltrate, revealing that in some cases the infiltrate of a particular cell type can be either a favorable or unfavorable indicator, depending on the class under study. This strengthens the growing idea that the immune infiltrate needs to be studied taking into account other variables and shows pathways and genes that deserve further investigation.

The differences observed between C1 and C2 also had implications in compound sensitivity. Gene set analysis predicted 255 compounds that could affect C1 and C2 differentially, and 70 drugs showed differential activity in GDSC cell lines. The main aim of performing such drug sensitivity analysis was to find pan-cancer patterns that could be used as a proxy for cancer therapy selection based on C1 and C2 profiles. While we could not find any clear sensitivity patterns when considering general mechanism of action (MoA) of anticancer drugs, we did have some intriguing findings. As expected, most type of cancers presented clear patterns of pan-resistance to therapies associated to C2 profiles (i.e., COAD/READ, ESCA, HNSC, KIRC). However, some cancers (i.e., DLBC, LAML) showed the opposite scenario: an increased sensitivity of C2 cells vs. C1 to several types of drugs. In addition, such situation was observed for particular drugs in cancers with the opposite pattern (i.e., parthenolide in ESCA). These findings suggest that these cancers cells have acquired actionable vulnerabilities to certain anticancer agents that could be exploited for successful treatment. Moreover, since C1 profiles are more similar to those of normal tissue, these findings also suggest the acquisition of such vulnerabilities should allow targeted treatment without major collateral effects on the normal cells of the patient. Future prospective studies using ex-vivo drug sensitivity screenings with patient-derived cells subjected to C1/C2 determination should help to validate these findings.

In summary, this study shows the use of a tissue-specific molecular signature as a tumor-agnostic classification tool with the aid of a proper uncertainty assessment method, resulting in consistent biological patterns. Our observations, added to the fact that PAM50 is an FDA approved signature for breast cancer, indicate that PAM50 could be repurposed for other cancer types with potential clinical implications that encompass drug sensitivity and immune infiltrate. This opens the door to studying any molecular signature that proved to be useful in a specific cancer type in other cancer types, which could result in a better understanding of this disease, more therapeutic options that could be offered, and improved patient stratification. A shiny app and R code to explore our findings in user-defined expression data is available at https://github.com/Dario-Rocha/tumor.agnostic.git.

## Supplementary Materials

The following are available online at https://www.mdpi.com/2073-4409/10/1/45/s1, Table S1: TCGA study abbreviations, Table S2: GDSC cell lines classification into TCGA cancer types, Table S3: C1/C2 PAM50 and C1/C2 classification in TCGA data, Table S4: C1/C2 classification by sample, Table S5: C1 vs. C2 differentially enriched gene sets in TCGA cohorts, Table S6: frequency of differentially expressed genes, Table S7: Enrichr pathway analysis results for the 95 core genes, Table S8: Enrichr results for the DSigDB gene sets with the 95 core genes, Table S9: Drug sensitivity analysis, Table S10: Survival models combining the C1/C2 classification and immune infiltrate.
